# Cardiac Tamponade and Primary Biliary Cholangitis: An Unusual Presentation and a Rare Association of Systemic Lupus Erythematosus

**DOI:** 10.7759/cureus.53501

**Published:** 2024-02-03

**Authors:** Ana Carvoeiro, Rita Mota, Patrícia Sobrosa, Alexandra Esteves

**Affiliations:** 1 Internal Medicine, Unidade Local de Saúde do Alto Minho, Viana do Castelo, PRT

**Keywords:** rare autoimmune disease, primary biliary cholangitis, pericardial effusions, cardiac tamponade, systemic lupus erythematosus

## Abstract

Systemic lupus erythematosus (SLE) is a disease known for its multiple manifestations, including numerous cardiac complications. While pericardial effusions are common in patients with SLE, cardiac tamponade is rare, and it is even rarer as an initial and isolated clinical manifestation of SLE. We describe a case of a young adult woman who presented with a four-week history of shortness of breath, orthopnea, and paroxysmal nocturnal dyspnea. Chest radiography revealed a significant increase in the cardiothoracic index, and transthoracic echocardiography confirmed a life-threatening cardiac tamponade that necessitated emergency pericardiocentesis and high-dose corticosteroids. Following a thorough investigation, we excluded viral infection, malignancy, tuberculosis, and other autoimmune diseases, and the patient was diagnosed with SLE based on the Systemic Lupus International Collaborating Clinics (SLICC) criteria. In this case report, we also present an uncommon association between SLE and primary biliary cholangitis (PBC). While both are autoimmune diseases, the coexistence of these two conditions in the same patient is rare. The report highlights the need for ongoing research to better understand the optimal management strategies for patients with coexisting autoimmune conditions.

## Introduction

Systemic lupus erythematosus (SLE) is a well-known autoimmune disease with diverse manifestations, capable of involving virtually any organ system and resulting in a broad range of clinical presentations. SLE is associated with numerous cardiac manifestations, including myocarditis, pericarditis, endocarditis, and conduction abnormalities [1]. While pericardial effusions are common in patients with SLE, cardiac tamponade is rare (1-2.5% incidence of tamponade in patients with SLE), and it is even rarer as the initial and isolated clinical manifestation of the disease [1-3].

We aim to describe an unusual presentation of this well-documented disease. Additionally, we want to emphasize that a prompt and thorough investigation of a patient presenting with a pericardial effusion is essential to prevent severe and potentially fatal complications.

In this case report, we also present an uncommon association between SLE and primary biliary cholangitis (PBC).

Several reports indicate that the incidence of coexisting PBC in patients with SLE is ≤2%, with results ranging from 0% to 2.7% [4]. Nowadays, it is uncertain whether some patients with concomitant SLE and PBC may have a common genetic susceptibility and/or immunological background favoring the development of these diseases [5]. A brief review of the literature regarding concomitant diagnosis of PBC and SLE revealed that PBC was diagnosed in first place in the majority (69%) of cases. It remains uncertain whether concomitant cases occur by chance or share a common immunological or genetic basis [6]. In our case, we also report the coexistence of these diseases in the same patient, a rare association, that not only broadens our understanding of the intricate interplay between distinct autoimmune conditions but also underscores the pressing need for further research and collaboration within the scientific community.

## Case presentation

A healthy 37-year-old Caucasian woman arrived at the emergency department, reporting a four-week history of worsening shortness of breath, orthopnea, and paroxysmal nocturnal dyspnea. She also experienced intermittent fever episodes during the same period, which subsided with paracetamol, loss of appetite, and progressive weakness, leading to multiple previous emergency department visits. On the day of admission, she reported pleuritic chest pain that worsened when supine. No other associated symptoms, such as joint pain, hair loss, oral ulcers, or rashes, were reported. She denied using any chronic medication. She recalled a hospitalization at age 16 due to toxic hepatitis, but there was no other relevant medical or family history. Upon physical examination, her blood pressure was 88/57 mmHg, and she had a respiratory rate of 28 breaths per minute and a heart rate of 55 beats per minute. The cardiorespiratory examination revealed noticeable jugular venous distension, muffled heart sounds, and decreased breath sounds in her lower lung fields. There was no evidence of joint inflammation, mucocutaneous lesions, peripheral swelling, or clubbing, and the remainder of the physical examination was unremarkable.

**Figure 1:**
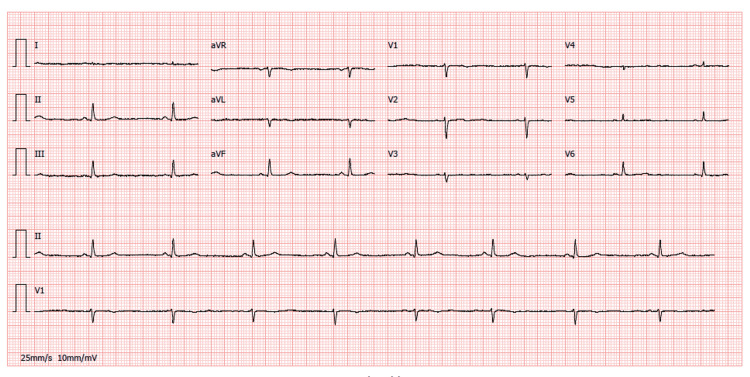
Electrocardiogram on admission revealing sinus bradycardia, low voltage, and electrical alternans

**Figure 2:**
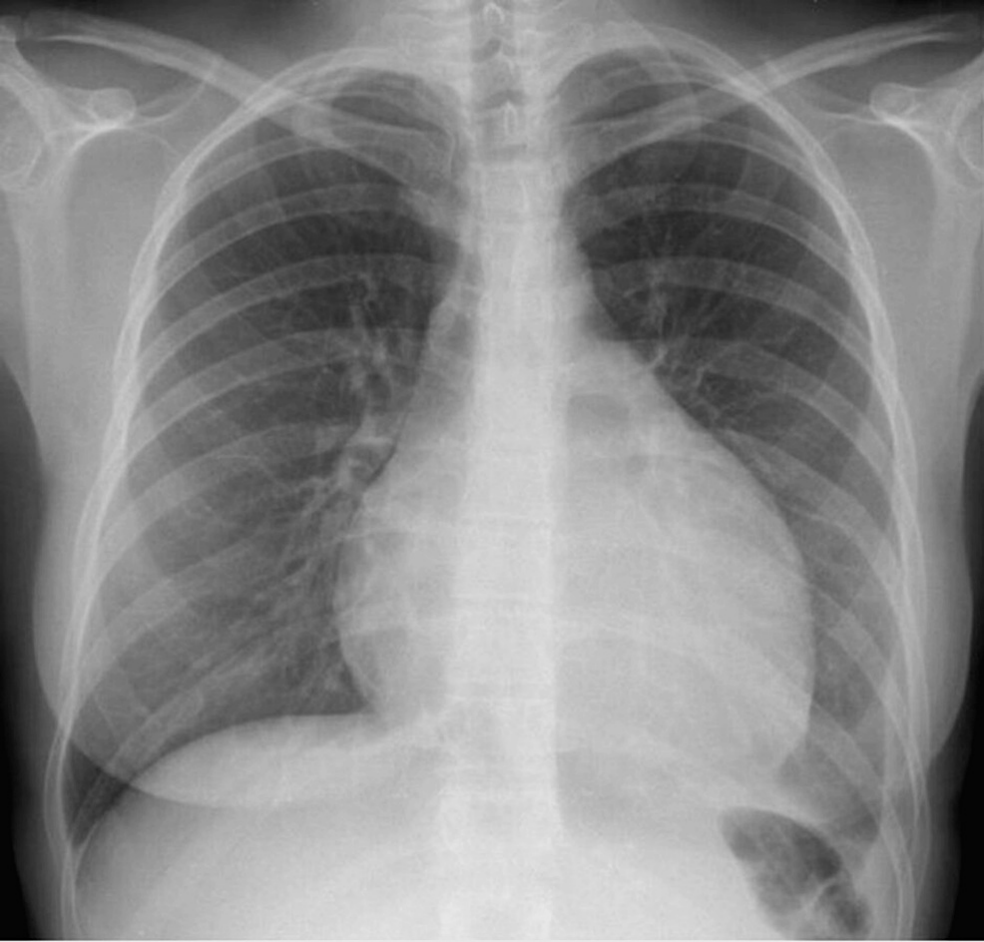
Chest radiography (posteroanterior view) at admission revealing enlarged cardiac silhouette and a small volume left pleural effusion

The electrocardiogram upon admission revealed sinus bradycardia, low voltage, and subtle electrical alternans in V4 (Figure 1). Chest radiography revealed an enlarged cardiac silhouette (Figure 2). Transthoracic echocardiography confirmed features of cardiac tamponade by revealing a large circumferential pericardial effusion (29 mm) with diastolic collapse of the right ventricle.

An emergency pericardiocentesis was performed, resulting in the immediate drainage of 900 ml of exudative fluid. The total cell count analysis showed 600/mm³ erythrocytes and 396/mm³ leukocytes, with a predominance of mononuclear cells (97/mm³). The adenosine deaminase (ADA) levels were low, measured at 27 U/L. The cytology of the pericardial fluid tested negative for malignant cells and no microorganisms were identified through Gram or Ziehl-Neelsen staining with further confirmation by a negative polymerase chain reaction (PCR) test for the detection of Mycobacterium tuberculosis. Additionally, the Mantoux skin test showed no reaction to tuberculin.

**Figure 3:**
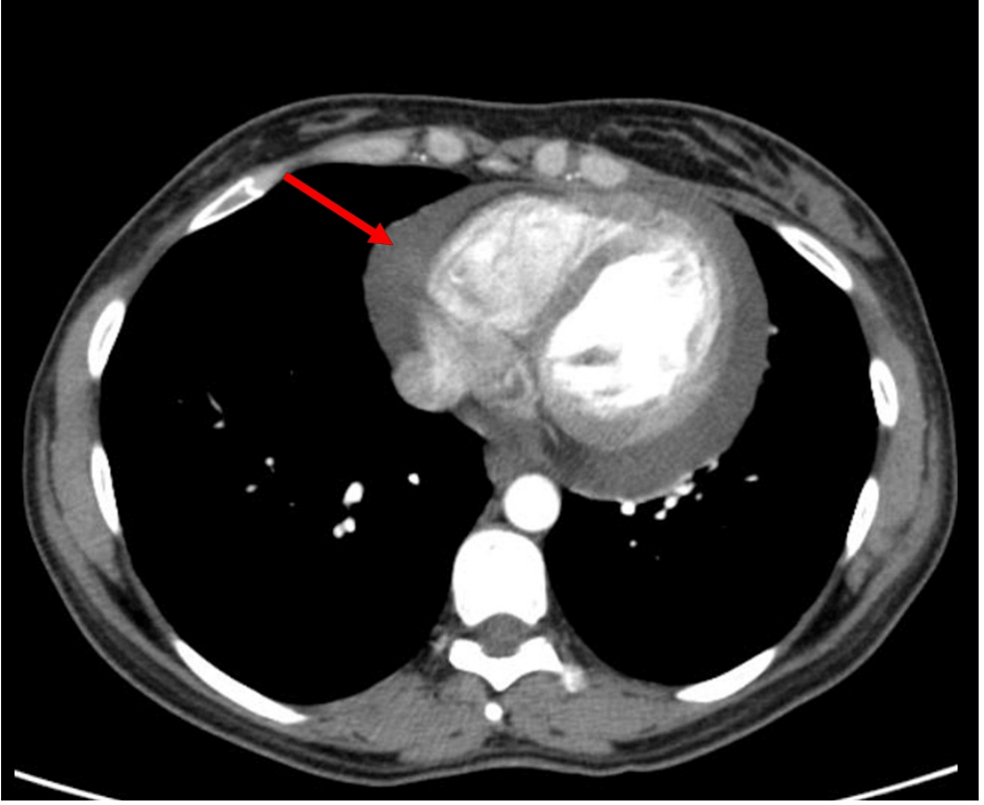
Thoracoabdominopelvic scan after pericardiocentesis showing moderate pericardial effusion (arrow)

A thoracoabdominopelvic CT scan was performed. The scan confirmed a pericardial effusion and revealed a minor left pleural effusion and minor ascites, with no suspicious nodules or masses (Figure 3).

Laboratory investigations indicated normocytic and normochromic anemia (hemoglobin 11.1 g/dL) and mild thrombocytopenia (103,000/L). Cardiac biomarkers, thyroid, liver (aspartate transaminase (AST) 27 UI/L; alanine aminotransferase (ALT) 30 UI/L; gamma-glutamyl transpeptidase (GGT) 40 UI/L; alkaline phosphatase (AP) 108UI/L))and kidney function tests, electrolytes, and blood glucose levels were within normal limits. Urinalysis showed no signs of hematuria, cellular casts, or proteinuria. Tests for HIV and hepatotropic viruses were negative.

Antinuclear antibodies (ANA) were present at a titer of 1:320. Concurrently, hypocomplementemia was identified (C3 of 60 mg/dL and C4 of 9.2 mg/dL), along with a positive direct Coombs test but without hemolytic anemia. Other immunological markers, including anti-Smith, antimitochondrial, antiphospholipid, anticardiolipin, anti-double-stranded DNA (dsDNA) antibodies as well as rheumatoid factor and extractable nuclear antigen (ENA) screen were all negative.

No uremic, viral, bacterial, or neoplastic causes of pericardial effusion were found. A diagnosis of SLE was ultimately established due to the presence of serositis, positive ANA, a positive direct Coombs test without hemolytic anemia, and hypocomplementemia. These findings met four of the 17 criteria proposed by the Systemic Lupus International Collaborating Clinics (SLICC).

**Figure 4:**
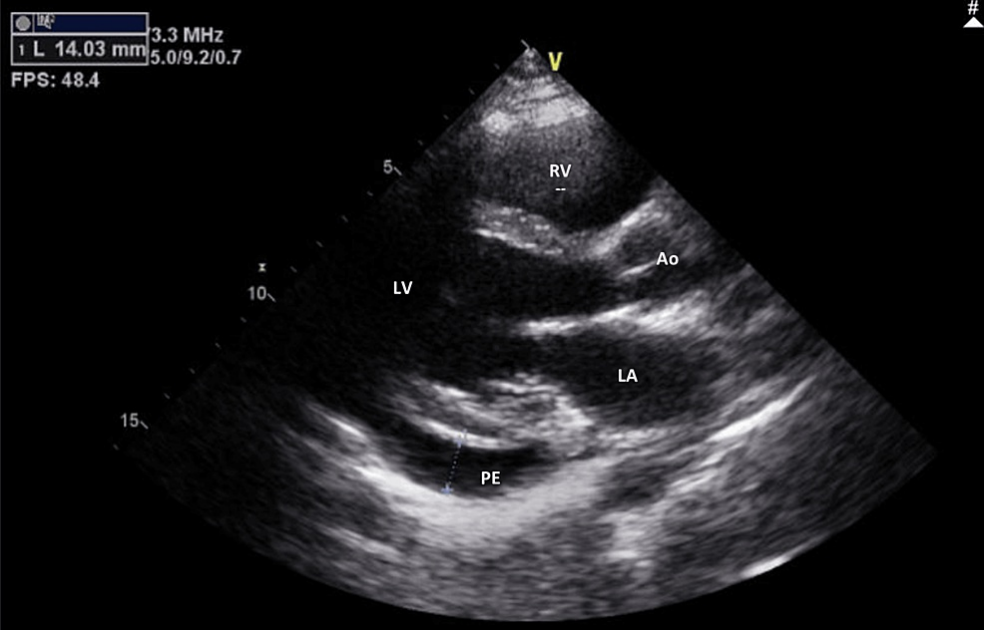
Echocardiogram (parasternal long-axis view) after pericardiocentesis demonstrating residual pericardial effusion of moderate volume PE: pericardial effusion; LA: left atrium; LV: left ventricle; Ao: aortic valve; RV: right ventricle

Pharmacological treatment was initiated with high-dose glucocorticoids at a dosage of 0.5 mg/kg/day. An echocardiogram was performed after pericardiocentesis, revealing the resolution of the right ventricle collapse and a decreased, but still significant, amount of pericardial fluid compared to the echocardiogram taken upon admission (Figure 4). Given these findings, an increase to 1 mg/kg/day of glucocorticoids was necessary due to the persistence of clinical and imaging features. Additionally, immunosuppression with azathioprine at a dosage of 50 mg/day was introduced, along with hydroxychloroquine at a dosage of 200 mg/day.

The patient was discharged on the nineteenth day of hospitalization for outpatient follow-up, denying any shortness of breath, chest pain, or cough. She was prescribed prednisolone 60 mg daily, azathioprine 50 mg daily, and hydroxychloroquine 200 mg twice daily.

**Figure 5:**
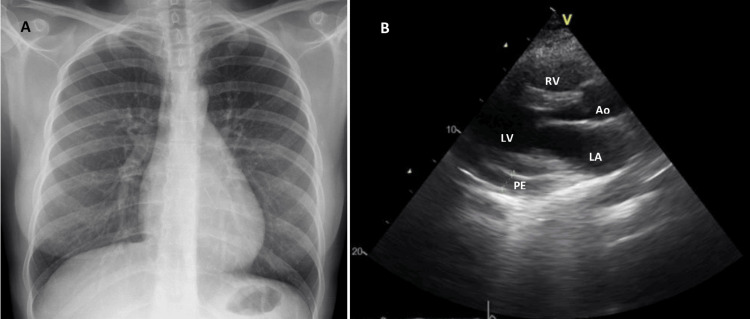
Follow-up chest radiography (A) and echocardiogram (B) revealing minor pericardial effusion PE: pericardial effusion; LA: left atrium; LV: left ventricle; Ao: aortic valve; RV: right ventricle

Chest radiography was repeated at follow-up, two months after discharge, and revealed a slight enlargement of the cardiac silhouette, although considerably improved (Figure 5) compared to the admission radiography (Figure 2). Transthoracic echocardiography also revealed minor and circumferential pleural effusion without echocardiographic signs of cardiac tamponade (Figure 5B). Prednisolone was progressively reduced and azathioprine was optimized to 100 mg/day.

Also, during the follow-up, the patient presented *de novo* a progressive increase of cholestasis parameters starting nine months after discharge (gamma-glutamyl transferase (GGT) - 103 UI/L<250 UI/L<430 UI/L; alkaline phosphatase (AP) - 103 UI/L<189 UI/L< 300UI/L), which motivated further study with abdominal ultrasound. This ultrasound revealed signs of chronic liver disease, prompting a review of the previous medical record. During her hospitalization at the age of 16 for toxic hepatitis, it was realized that the hypothesis of autoimmune hepatitis had been considered, but this was not confirmed by findings after liver biopsy. A new immunological study was performed, including a positive anti-centromere antibody and negative antimitochondrial, smooth muscle, anti-LCI, and anti-LKM antibodies. A liver biopsy was repeated, and it was compatible with PBC type 1, showing lymphocytic infiltration surrounding the bile duct, with focal infiltration of the duct wall, associated with fibrosis.

The patient started treatment with ursodeoxycholic acid 500 mg bid. However, there was an unfavorable clinical evolution over a period of three years, with the development of liver cirrhosis, the need for weekly evacuative paracentesis, and hospitalizations for upper digestive hemorrhage in the context of esophageal varices. She was considered for a transjugular intrahepatic portosystemic shunt (TIPS) procedure while awaiting a liver transplant. Unfortunately, she passed away due to hemorrhagic shock during the procedure.

## Discussion

SLE is a well-known chronic multisystem disorder. Immune-complex depositions in SLE result in an inflammatory reaction that can affect any organ system. Patients may present with a wide array of symptoms, signs, and laboratory features, and their prognosis varies depending on disease severity and the type of organ involvement [1]. Cardiac manifestations are reported to be one of the main complications contributing to the morbidity and mortality of patients with this disease [1-2]. While pericardial involvement is a well-recognized cause of symptomatic cardiac disease in almost half the patients, cardiac tamponade is an uncommon manifestation, with reports varying from 1% to 22% of patients [1,3]. In this case report, cardiac tamponade was not only the initial presentation of SLE but also the sole manifestation of the disease, contributing to a delayed diagnosis.

Cardiac tamponade is a medical emergency that develops when the volume of pericardial effusion reaches a critical amount, leading to hemodynamic compromise due to increased intra-pericardial pressure preventing cardiac chamber filling [7]. Symptoms of SLE-related pericarditis and cardiac tamponade can be non-specific and initially overlooked by both patients and physicians, as described in this case report. The most common presenting symptoms of tamponade among patients described in the literature were shortness of breath, chest pain, and fever [7], all of which were present in our patient. However, other symptoms referable to increased filling pressures and limited cardiac output can be observed, including peripheral edema and fatigue.

Establishing the diagnosis of SLE can be challenging due to its clinical heterogeneity, which can lead to a significant diagnostic delay. Diagnosis is based on clinical judgment and supported by immunologic features, after excluding alternative diagnoses. Classification criteria for SLE were developed in 1997 by the American College of Rheumatology (ACR) [8,9] and revised in 2012 by the SLICC [10]. According to this classification, a diagnosis of SLE is established if the patient meets at least 4 of the 17 total criteria, with at least one clinical and one immunologic criterion. Alternatively, biopsy-proven lupus nephritis associated with positive ANA or anti-dsDNA antibodies is also diagnostic of SLE. Our patient met 4 of the 17 criteria proposed by SLICC. However, establishing the diagnosis was a challenge, primarily due to the exceptional disease presentation and the absence of other common clinical syndromes associated with SLE such as renal, cutaneous, and/or musculoskeletal manifestations. For that matter, a thorough investigation was of paramount importance in diagnosing SLE. Simultaneously, other common etiologies of tamponade such as bacterial, tuberculous, malignant, and uremic pericardial effusion [11] were ruled out by clinical and laboratory tools, including Gram stain, culture, cytology, PCR, and biochemical testing. This exclusion was mandatory in a country with a high prevalence of tuberculosis, where this disease remains one of the leading causes of pericardial effusion, following idiopathic and neoplastic etiologies.

In this case report, we present an uncommon association between SLE and PBC. While both are autoimmune diseases, the coexistence of these two conditions in the same patient is rare. The diagnosis of PBC in this patient was made later in the course of her illness. Earlier recognition and diagnosis of PBC could have potentially allowed for earlier intervention and management of this condition. This case underscores the importance of considering multiple diagnoses when faced with complex clinical presentations, the need for careful and thorough medical history taking, and the complex and multifaceted nature of autoimmune diseases.

The patient’s cardiac tamponade was attributed to SLE and not PBC. The treatment of choice included drainage by emergency pericardiocentesis and high-dose glucocorticoids. Corticosteroids are not typically used in the management of PBC, and their use in this case was specifically targeted at controlling the patient’s SLE. The addition of disease-modifying antirheumatic drug therapy (DMARD) such as azathioprine, cyclophosphamide, mycophenolate mofetil, or methotrexate depends on the severity of the disease and whether the patient has additional organ-threatening disease. Antimalarial drugs (hydroxychloroquine and chloroquine) have shown additional benefits, including a decrease in serositis recurrences [12,13].

In our patient, azathioprine was used as part of the treatment regimen for SLE, not PBC. It’s important to note that while azathioprine can have side effects, there’s no evidence to suggest that it negatively affected the prognosis of PBC in this patient. The progression of PBC to cirrhosis in this patient is likely due to the natural course of the disease rather than the use of azathioprine. Some studies suggest a beneficial effect of azathioprine in PBC [14] while others found no evidence to support its use for patients with PBC. According to a review by Cochrane, azathioprine has no effect on survival, itching, progression of the disease (cirrhosis development), or quality of life in PBC patients [15].

## Conclusions

This clinical report highlights the need to consider LSE in the differential diagnosis of patients presenting with isolated cardiac tamponade. In this particular case, the early recognition of the cardiac tamponade and its exhaustive further investigation allowed a prompt diagnosis of LSE. Treatment with pericardiocentesis drainage and early glucocorticoid administration is of the utmost importance in order to prevent precarious morbimortality associated with this disease.

This case also underscores the importance of individualized treatment plans based on the patient’s specific diagnoses and overall health status. It also highlights the need for ongoing research to better understand the optimal management strategies for patients with coexisting autoimmune conditions.
